# New insights into spontaneous pneumothorax: A review

**DOI:** 10.7196/AJTCCM.2021.v27i1.054

**Published:** 2021-03-09

**Authors:** E H Louw, J A Shaw, C F N Koegelenberg

**Affiliations:** Division of Pulmonology, Department of Medicine, Stellenbosch University and Tygerberg Academic Hospital, Cape Town, South Africa

**Keywords:** Pneumothorax, Spontaneous

## Abstract

A spontaneous pneumothorax is a pneumothorax that does not arise from trauma or an iatrogenic cause. Although the traditional classification of either primary or secondary spontaneous pneumothorax based on the absence or presence of overt underlying lung disease is still widely used, it is now well recognised that primary spontaneous pneumothorax is associated with underlying pleuropulmonary disease. Current evidence indicates that computed tomography screening for underlying disease should be considered in patients who present with spontaneous pneumothorax. Recent evidence suggests that conservative management has similar recurrence rates, less complications and shorter hospital stay compared with invasive interventions, even in large primary spontaneous pneumothoraces of >50%. A more conservative approach which is based on clinical assessment rather than pneumothorax size can thus be followed during the acute management in selected stable patients. The purpose of this review is to revisit the aetiology of spontaneous pneumothorax, identify which patients should be investigated for secondary causes and to give an overview of the management strategies at initial presentation as well as secondary prevention.

## Background

Pneumothorax is a composite word of Greek origin derived from πνεύμα (pneuma) and θώραξ (thorax), meaning air in the thorax and specifically within the pleural space. The term was first used by a French physician Jean Marc Gaspard Itard, who was a student of René Laennec, the inventor of the stethoscope, in the 19th century.^[1]^ During that period, the most common cause of a pneumothorax was tuberculosis. Indeed, the iatrogenic introduction of a pneumothorax in the treatment of tuberculosis was accepted clinical practice in the late 19th century and continued until the 1950s, when the use of anti-tuberculosis treatment became widely available.^[2]^

The term spontaneous pneumothorax refers to a pneumothorax arising from neither trauma nor an iatrogenic cause. The traditional classification of either primary spontaneous pneumothorax (PSP) or secondary spontaneous pneumothorax (SSP) distinguishes between pneumothoraces with (secondary) and without (primary) prior known or clinically apparent underlying lung disease. However, although it is still widely used, the utility of making such a distinction is being challenged in many circles.

The purpose of this narrative non-systematic review is to highlight selected emerging evidence in this field and guide the practising clinician on an evidence-based approach to the management of spontaneous pneumothorax. For the purposes of the review, we consider the single entity of spontaneous pneumothorax and specify PSP or SSP only where appropriate to the literature being referenced.

## Aetiology

Several risk factors for spontaneous pneumothorax have been identified (Table 1). Individuals who present with a pneumothorax as a first manifestation of their lung disease tend to be tall with a low body mass index, but PSP is most strongly associated with tobacco smoking.^[3]^ Cannabis smoking, altitude and air pollution are additional risk factors.^[3–5]^ In females, a rare cause is catamenial pneumothorax.^[6,7]^ The peak incidence for PSP occurs at 35 years of age, whereas SSP occurs later in life at 53 years, reflecting a parallel increase in chronic lung disease as age increases.^[8]^ The traditional SSP is associated with overt structural lung disease of which the most common underlying cause is chronic obstructive pulmonary disease.^[9,10]^ Although the exact prevalence is not known, it is well recognised that in regions with high tuberculosis (TB) and HIV burden like South Africa (SA), infectious causes such as *Pneumocystis jirovecii* are the common causes of spontaneous pneumothorax.^[11]^

It is now recognised that PSP is caused by underlying structural lung abnormalities that are not visible on a routine chest radiograph and not clinically apparent prior to the presentation with a pneumothorax. Abnormalities which have been detected with computed tomography (CT) or on histopathology include emphysema-like changes (blebs and bullae) of lung parenchyma under the visceral pleura as well as a diffuse decrease in lung density measured by CT. One study identified a diffuse inflammatory process in the underlying lung parenchyma with subsequent increase in the porosity of the visceral pleura,^[12]^ and another found fibroblastic lesions consisting of pleural fibrosis with islands of fibroblastic foci within a myxoid stroma.^[13]^ Numerous genetic syndromes have been associated with spontaneous pneumothorax (Table 1) and therefore a detailed medical and family history, and careful clinical assessment of the patient should be performed.

## The role of imaging

Routine CT scanning was traditionally not advocated after the first episode of a perceived PSP. A recent clinical review of genetic abnormalities in PSP has suggested that a CT scan should be performed in patients with a family history of pneumothorax, lung blebs, cysts, bullae or physical examination suggestive of a syndrome.^[14]^ Some 10 - 12% of patients with PSP have a family history of a pneumothorax and it is thought that they have a higher recurrence rate.^[15,16]^ It is reported that between 5 - 10% of patients with an apparent PSP have underlying Birt-Hogg-Dubé (BHD) syndrome.^[17,18]^ In this cystic lung disease, there is a clear cost-benefit of performing a chest CT to identify patients with a high risk of recurrent pneumothorax and numerous other long-term health implications.^[19]^

It has also been suggested that a CT be performed in females with a first episode of spontaneous pneumothorax to diagnose occult lymphangioleiomyomatosis (LAM), as new advances in the treatment of this condition have emerged.^[20]^ In a study from Taiwan,^[21]^ 3.6% of the patients had an unexpected finding on CT scan that was not seen on the chest radiograph and the majority of them were females.

CT may identify a population at higher risk for recurrence of PSP through the assessment of the severity of the underlying abnormalities of the visceral pleura, but this remains to be proven in prospective studies.^[22]^

A CT scan should thus be considered in the following patients with spontaneous pneumothorax: patients older than 55 years of age as an underlying lung disease is more likely; patients with a family history of pneumothorax, lung blebs, cysts or bullae; patients with a family history or clinical signs of a genetic syndrome; females; and non-smokers.

## Management of a spontaneous pneumothorax

### Does size matter?

**Fig. 1:**
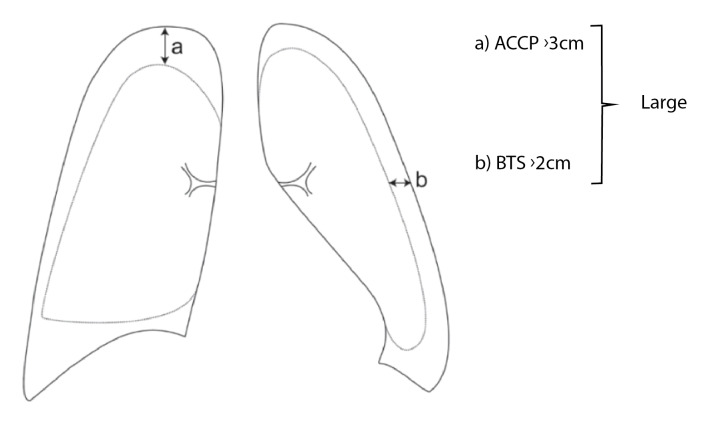
Estimation of size of a pneumothorax based on routine chest radiography. The American guidelines generally use the intrapleural distance from the apex to the cupola of the lung (a), whereas the British Thoracic Society uses the intrapleural distance at the hilum (b).

Current guidelines for the management of spontaneous pneumothorax require an assessment of the size of the pneumothorax.^[23,24]^ However, the evidence for using size in the management of pneumothoraces is not robust and there is poor agreement in the methods of measurement.^[25]^ CT is generally acknowledged as the best method for estimating the size of a pneumothorax by various techniques including the Collins or Rhea methods.^[26]^ The traditional chest radiograph-based method of quantification is the light index, but its accuracy is inconsistent.^[3,27]^ The British Thoracic Society (BTS) guidelines use a measurement of the distance from the chest wall to the lung edge taken at the level of the hilum on chest radiograph, while the American College of Chest Physicians (ACCP) measure this distance at the apex of the lung (Fig. 1).^[23,25]^ A large pneumothorax is more than 2 cm at the hilum according to the BTS and more than 3 cm at the apex according to the ACCP. Nikolic *et al.*^[28]^ elegantly demonstrated that the use of the BTS guidelines is associated with less invasive treatment than the ACCP guidelines and that following the ACCP guidelines resulted in 65% of the patients having an intercostal drain (ICD) inserted unnecessarily.

The BTS guidelines do state that the size of the pneumothorax is less important than the degree of clinical compromise. The decision to proceed with invasive management should rather be based on symptoms and the clinical stability of the patient. They defined stability as having a respiratory rate <24, heart rate of 60 - 120 bpm, oxygen saturation more than 90% on room air, blood pressure >90/60 mmHg and being able to complete full sentences between breaths.^[23,24]^ While these criteria are not absolute, the decision to pursue a non-invasive management strategy in a pneumothorax which exceeds the BTS and ACCP size-based guidelines must be supported by an initial clinical assessment of stability as well as close observation.

### Needle aspiration

International guidelines disagree on the role of needle aspiration. The ACCP guidelines do not advocate needle aspiration if active intervention is required and the BTS guidelines advise needle aspiration of up to 2.5 L as the first step in the management of PSP, with the option of proceeding to catheter drainage with a small-bore ICD. Needle aspiration has been shown to be effective, with decreased length of hospital stay, decreased complications and similar recurrence rates compared with ICD.^[29–32]^ However, needle aspiration fails if the air leak is still present at the time of the procedure. It has been shown that compliance with the BTS guidelines in general is poor and most clinicians favour ICD insertion as primary intervention over needle aspiration.^[33,34]^ This might reflect the fact that 20 - 50% of patients will require a second procedure despite the needle aspiration,^[29,35]^ which physicians are keen to avoid. There is also a perception that needle aspiration is more time-consuming in the emergency unit setting.^[32]^

In general, we would recommend that in patients with spontaneous pneumothorax who are judged to need intervention, are not *in extremis*, and the risk of persistent air leaks is judged to be low, needle aspiration may be attempted first. If needle aspiration is not possible or has failed, it is advised to insert a small-bore ICD.

### Rethinking intercostal drain use

For many years, it has been argued that in addition to allowing the air in the pleural space to escape and the lung to re-expand after a pneumothorax, an ICD also causes inflammation of the pleural surface and promotes auto-pleurodesis, which reduces the relapse rate.^[36]^ However, there is a growing body of evidence suggesting that a conservative approach to managing pneumothoraces which avoids ICD use may have better outcomes in selected patients, with fewer infectious complications, bleeding, organ injury, shorter hospital stay and lower subsequent risk of recurrence.

The average rate of resolution of a pneumothorax without the insertion of an ICD ranges from 1.25% to 2.2% of the volume of the hemithorax per day, although larger pneumothoraces tend to resolve faster than small ones and there is significant variation between individuals.^[23,25]^ Theoretically, the longer time that the lung spends partially collapsed allows the pleural defect to heal. The level of evidence for supplemental oxygen treatment during conservative management is low.^[37]^ One study found a rate of resolution of 4.27% per day with supplemental oxygen compared with 2.06% per day without.^[38]^

As early as 1966, Stradling and Poole^[39]^ published a large series of patients who were managed conservatively and found that only 25% of all spontaneous pneumothoraces needed any form of active intervention. In the group without underlying lung disease, 80% expanded without any intervention, with a mean expansion time of 22.5 days and the mean expansion time was 30.8 days without any intervention in the group with underlying emphysema. More than half of the patients considered to have underlying emphysema were managed without any intervention. Interestingly, the relapse rate was 11% over a period of 6 years, which is strikingly lower than the recently documented relapse rates of 22 - 54% at 1 year in patients managed actively with aspiration or ICD insertion.^[30,40-42]^

Similarly, a recent Australian^[43]^ retrospective study found that conservative management of PSPs (irrespective of size) had similar recurrence rates, fewer complications and shorter hospital stay than the intervention group, even in large pneumothoraces of >50% of the hemithorax. This study however did not include patients with overt underlying lung disease.

A landmark study conducted by Brown *et al.*^[44]^ comparing conservative with interventional management of moderate-to-large PSP showed an almost twice as high recurrence rate in the interventional group (16.8% v. 8.8%), along with decreased length of hospital stay and complications in the conservative arm. Less than a quarter (15.4%) of patients initially selected for conservative management required intervention during the initial follow-up due to persistent symptoms or instability. This trial provides more evidence that conservative management can be considered even in large pneumothoraces provided that the patient remains haemodynamically stable and has prompt access to healthcare.

In addition to rethinking whether an ICD is indicated in the first instance, one should also carefully consider the size of the ICD that is inserted. The current BTS guidelines show that small ICDs of 16F or less results in reduced complication rates compared with large-bore ICDs>16F.^[23]^ Moreover, a more recent meta-analysis concluded that ICDs of 14F or less have lower complication rates, similar success rates, shorter drainage duration and shorter length of hospital stay.^[45]^ In the majority of medical and emergency wards in SA, the common practice is to insert an ICD for any pneumothorax and the only available ICDs are 24F and above. It is therefore important to emphasise that both patient safety and patient comfort are improved with the thoughtful use (or not) of an ICD.

### Ambulatory management

**Fig. 2:**
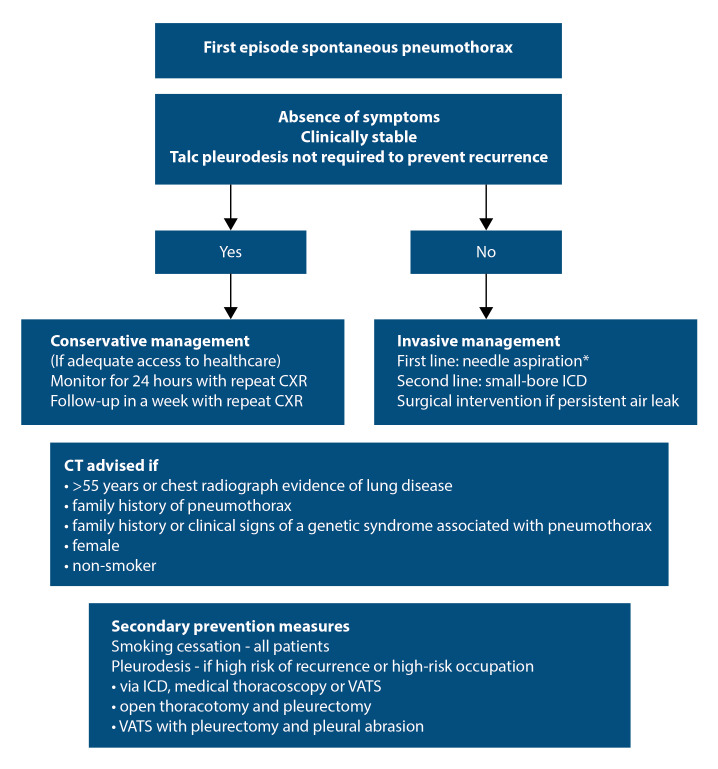
Proposed algorithm for management of primary spontaneous pneumothorax. CXR = chest radiograph ICD = intercostal drain CT = computed tomography (chest) VATS = video-assisted thoracoscopic surgery. *Not if talc pleurodesis via ICD is planned.

There may be a role for the outpatient management of a spontaneous pneumothorax in the correct setting. Two studies recently evaluated ambulatory management of spontaneous pneumothoraces with the placement of small-bore ICD’s attached to one-way valves which allowed slow air leakage. The one study had a success rate of 79% in patients with large pneumothoraces and 37% had full outpatient management,^[46]^ while the other study found that ambulatory management can be effective even in patients with overt underlying lung disease with a mean drainage time of 5.84 days.^[47]^ Both studies found that this method was associated with reduced hospital costs and avoided potential tension pneumothoraces. In a situation where a patient has ready access to transport and is close to the treating healthcare facility, ambulatory management may be an appropriate strategy. An algorithm for the management of a first episode of spontaneous pneumothorax (primary or secondary) is suggested (Fig. 2).

## Preventing recurrences

The greatest risk for recurrence is within the first year,^[24,42]^ with a recent systematic review finding that the pooled 1-year recurrence rate for PSP is 29%.^[40]^ Risk factors for recurrence include smoking, younger age, female sex, low body weight, height and radiological evidence of underlying lung abnormalities.^[40,48]^ Cessation of smoking is central to preventing recurrence and physicians must be active in advocating this for all their patients.^[49]^

Therapeutic options for preventing recurrence of a spontaneous pneumothorax include pleurodesis with large-particle talc or other sclerosants via medical thoracoscopy or video-assisted thoracoscopic surgery (VATS), open thoracotomy and pleurectomy, and VATS with pleurectomy and pleural abrasion. Open thoracotomy and pleurectomy has a recurrence rate of 1% while VATS with pleurectomy and pleural abrasion has a recurrence rate of 5%.^[50]^ However, VATS is associated with shorter hospital stay, reduced hospital costs, postoperative bleeding complications and pain than open thoracotomy.^[51–53]^ Guidelines advise definitive pleurodesis in patients with recurrent PSP, persistent PSP, bilateral PSP, professions at risk and in patients with underlying overt lung disease.

Once patients have undergone initial management of the pneumothorax, whether treated invasively or conservatively, the risk for recurrence should be assessed. The management strategy for prevention will depend on available expertise, operative risk and patient preference. In most cases, VATS or medical thoracoscopy is preferred and for those unable or unwilling to undergo surgery, chemical pleurodesis via ICD is recommended. Ideally, the timing of the procedure should be within the same hospital admission as the risk for recurrence is highest within the first month,^[23]^ although this is not always logistically possible.

## Conclusion

Our knowledge about spontaneous pneumothoraces has evolved in recent years, and it is clear that the old labels of primary and secondary may not be appropriate anymore. Individuals previously managed under the PSP guidelines invariably have pleuropulmonary disease on chest CT or histology and the literature is still unclear on how we should deal with this fact. Evidence seems to suggest that more patients deserve to be investigated for underlying disease than is currently recommended. In addition to this, there are emerging data to suggest that conservative treatment of spontaneous pneumothoraces of any size is appropriate in stable patients. However, careful consideration must be given to the necessity to intervene to prevent recurrence, especially in settings where access to VATS or open pleurectomy is limited and talc pleurodesis is the only practical option. If initial invasive management is needed, it need not always be the traditional large-bore ICD. In fact, a small-bore ICD is preferable and ambulatory management could be considered. The next decade should see randomised trials to clarify these issues. For now, clinicians should endeavour to be more critical of intervention choices in a patient with a spontaneous pneumothorax.

## Figures and Tables

**Table:** Table 1. Risk factors for a spontaneous pneumothorax

**Risk Factor**	**Mechanism**
**Tall and thin**	Greater distending pressures in apex predispose to the development of apical subpleural blebs.
**Smoking**	Airway inflammation and respiratory bronchiolitis.
**Underlying lung disease**	Structural lung abnormalities and altered airflow dynamics.
COPD	
TB/infection	
Interstitial lung disease	
Malignancy	
Cystic fibrosis	
**Other**	
Catamenial pneumothorax	Pleural endometriosis, circulating endometrial cells, transdiaphragmatic passage of air during menstruation, hormonally mediated vascular and bronchiolar vasoconstriction.
**Genetic syndromes**	
Syndromes related to tumour-suppressor genes	Rupture of subpleural parenchymal cysts.
*Birt-Hogg-Dubé syndrome*					
*Tuberous sclerosis*	
*Pulmonary LAM*	
Connective tissue diseases	Defects in structure of visceral pleura.
*Marfan syndrome*	
*Ehlers-Danlos syndrome*	
*Loeys-Dietz syndrome*	
*Homocysteinuria*	
Normal lung architecture effaced	Structural abnormalities and altered airflow dynamics.
*Alpha-1 antitrypsin deficiency*	
*Cystic fibrosis*	

COPD = chronic obstructive pulmonary diseases

LAM = lymphangioleiomyomatosis
